# Antibacterial Activity of *Lactobacillus* Strains Isolated from Mongolian Yogurt against *Gardnerella vaginalis*

**DOI:** 10.1155/2020/3548618

**Published:** 2020-04-22

**Authors:** Zhixiang Qian, Dan Zhao, Yu Yin, Hui Zhu, Daijie Chen

**Affiliations:** College of Pharmacy, Shanghai Jiaotong University, Shanghai 201100, China

## Abstract

Worldwide interest in the use of functional foods containing probiotic bacteria such as *Lactobacillus* and *Bifidobacterium* for health promotion and disease prevention has increased significantly. Probiotics have demonstrated beneficial properties including strengthening the body's natural defense system, inhibiting the growth of pathogenic bacteria, and regulating mental activity, but their effects on the human vagina have not been fully elucidated. The primary purpose of our study was to isolate *Lactobacillus* strains from old yogurt, a traditional dairy product, and investigate their probiotic potential with respect to the human vaginal system. Four *Lactobacillus plantarum* (*L. plantarum*) strains, named ZX1, ZX2, ZX27, and ZX69, were isolated from the yogurt samples. Simultaneously, we used a commercial *Lactobacillus* strain (*Lactobacillus delbrueckii* DM8909) as a control strain. We tested the antimicrobial activity of *Lactobacillus* isolates against *Escherichia coli* and *Gardnerella vaginalis* by agar spot and well diffusion tests. Then, we tested the antibiotic susceptibility of the 5 strains by using the minimal inhibitory concentration method. We attempted to detect possible bacteriocin genes by PCR sequencing technique. Using a chemically defined medium simulating genital tract secretions, we found that the selected *Lactobacillus* strains could alter the expression of known virulence genes in *Gardnerella vaginalis.* Bacteriocins derived from these isolated strains had potent antibacterial activity against *G*. *vaginalis* and *E*. *coli*, with the most effective activity observed in the case of ZX27. In addition, all strains including the *L*. *delbrueckii* DM8909 were positive for the presence of the plantaricin cluster of genes described in *L*. *plantarum* C11. The tested stains possessed the *pln* gene indicating that one of the antibacterial agents was plantaricin. We assume that the production of antimicrobial substances such as bacteriocins induce *G. vaginalis* to upregulate antimicrobial resistance genes. The new isolated strains have bacteriocin-related genes and can change the antimicrobial resistance gene transcription of *G*. *vaginalis*.

## 1. Introduction

Bacterial vaginosis (BV) is the most common cause of abnormal vaginal discharge in women of child-bearing age [1, 2]. Although antimicrobial drugs, such as clindamycin and metronidazole, are recommended for the treatment of BV, the use of these drugs is limited due to their side effects such as selecting of antibiotic-resistant pathogenic strains or causing superinfection [3]. Healthy vaginal flora is dominated by *Lactobacillus* that can lower the vaginal pH and/or produce metabolites such as hydrogen peroxide, lactic acid, and antibacterial molecules, including bacteriocins [4]. *Lactobacillis* plays a vital role in maintaining vaginal health. *Lactobacillus* species can act as biomarkers and agents that can promote various aspects of vaginal health [5]. *G. vaginalis*, a facultative anaerobe, is a leading harmful bacteria in BV [6]. Many studies have been carried out to investigate the dynamics between *Lactobacillus* and *G*. *vaginalis in vitro* and *in vivo*. The common characteristics of BV patients are the decrease of *Lactobacillus* and the increase of *G*. *vaginalis* in the vagina. *L. rhamnosus* GR-1 was the first probiotic used as a vaginal microbiota regulating *Lactobacillus* in 1988 [7]. It was demonstrated that *L*. *crispatus* could repress the expression of *vly* and *sld* genes in *G*. *vaginalis* [8, 9]. Other researchers discovered that *Lactobacillus* could kill *G*. *vaginalis* and inhibit NF-*κ*B activation, *G*. *vaginalis*-induced epithelial cell disruption, myeloperoxidase activity, and IL-1*β* and TNF-*α* expression in mice [10, 11]. Clinical experiments also indicated that probiotics such as *L*. *rhamnosus* GR-1 and *L*. *reuteri* RC-14 have a therapeutic effect in reproductive tract infection [12]. *L*. *plantarum* is a common species in dairy products. Some researchers also reported that they have isolated *L*. *plantarum* from cheeses [13, 14] and that these isolates have an antibacterial activity against *L*. *monocytogenes*, *E*. *coli*, and *S*. *enteritidis*. Devi et al. [15] isolated *L*. *plantarum* subsp. *plantarum* MTCC 5422 from fermented cereal and discovered that MTCC 5422 effectively induced inflammatory conditions due to its anti-inflammatory activity. Except for *G*. *vaginalis*, *E*. *coli* remains the most common etiological organism for causing urinary tract infections (UTIs) [16].

Bacteriocins are small, heat-stable peptides which are produced by bacteria and are active against other bacteria [17]. There are two antibacterial mechanisms of bacteriocins: binding to lipid II and preventing cell wall synthesis, which can lead to cell death, or using lipid II as a docking molecule to initiate a process of membrane insertion and pore formation that leads to rapid cell death [18]. Bacteriocins have also been shown to be involved in immunoregulation. It was demonstrated that plantaricin EF produced by *L*. *plantarum* NCIMB8826 elevated TNF-*α* and IL-6 concentrations in mouse intestines [19].

Old yogurt is a traditional fermented food for herdsmen in Inner Mongolia, China, which is a solid yoghurt, and has longer fermentation process and more sour taste than the regular yogurt. Old yogurt only contains milk, sugar, or honey and a fermented culture. Yogurt is an important source of probiotics and has multiple beneficial effects on human health. For example, *Lactobacillus bulgaricus* was isolated from yogurt in 1905 [20]. Zhu et al. [21] showed that bioyogurt and the probiotics that it contains were capable of inhibiting specific periodontal pathogens. Yogurt with selected probiotic strains such as N1115 may reduce the risk of acute upper tract infections in the elderly [22].

The aim of our study was to isolate *Lactobacillus* strains from old yogurt and identify whether they had any potential activity against *Gardnerella vaginalis*.

## 2. Materials and Methods

### 2.1. Sample Collection, Strains, Cell Lines, and Growth Conditions

A total of 10 condensed yogurts were purchased from the shepherds in the Hulun Buir region of China. Then, the yogurts were shipped by cold-chain transportation to Shanghai Jiaotong University, Shanghai, for further experiments.

We grew *Lactobacillus* strains in de Man, Rogosa, and Sharpe (MRS) medium for 16–24 h at 37°C in an anaerobic system (RUSKINN, UK). *Gardnerella vaginalis* (ATCC49145) was purchased from the Guangdong culture collection center and cultured in brain-heart infusion (BHI) broth supplemented with yeast extract (1%), maltose (0.1%), glucose (0.1%), and horse serum (10%) (BHIS) at 37°C for 24 h under anaerobic conditions. *Escherichia coli* (ATCC 25922) was grown on Luria-Bertani medium (LB) for 12 h at 37°C. Hela cells, a cervical epithelial cell line, were grown in Dulbecco's Modified Eagle's Media (DMEM) (HyClone, USA) supplemented with 10% fetal bovine serum (FBS) (Gibco, USA) and 1% antibiotics (penicillin and streptomycin) (Gibco, USA) at 37°C in a 5% CO_2_ atmosphere.

### 2.2. Isolation and Identification of Lactobacillus Strains

MRS agar and broth were used to streak yogurt samples and enrich for the *Lactobacillus* strains. Colonies with typical *Lactobacillus* morphology, white in color and producing a fruity aroma, that were Gram positive were selected and inoculated in MRS broth. Ultimately, isolates were confirmed and identified by genetic analysis using PCR and 16S rDNA sequencing. The genomic DNA of the *Lactobacillus* strains (from 2 mL of *Lactobacillus* culture) was extracted using a bacterial DNA isolation kit (Sangon Biotech, China). Universal PCR primers 27F (AGAGTTTGATCCTGGCTCAG) and 1492R (TACGGCTACCTTGTTACGACTT) were used to amplify the 16S rDNA gene. The PCR protocol was performed with the following thermal cycling parameters: 95°C for 10 min followed by 30 cycles of denaturation at 94°C for 30 S, primer annealing at 60°C for 30 s, elongation at 72°C for 1.5 min, and thermal retardation at 72°C for 10 min. Further, the PCR products were sequenced at the Invitrogen Biotechnology Company (Shanghai, China) and subjected to Basic Local Alignment Search Tool (BLAST) in the National Center for Biotechnology Information (NCBI). Finally, the phylogenic tree was constructed using the neighbor-joining method with MEGA 7 software using a bootstrap value of 1000. A commercial strain, *Lactobacillus delbrueckii* DM8909, isolated from Dingjunsheng (live *Lactobacillus* capsule for vaginal use®, Wanze Shuangqi) was used as the control.

### 2.3. Antibacterial Tests In Vitro by Agar Spot and Well Diffusion Tests

The antagonistic activity of the *Lactobacillus* strains against *G. vaginalis* and *E*. *coli* was investigated as previously described [23], with slight modification. In brief, for the spot agar test, a 2 *μ*L aliquot from each *Lactobacillus* strain suspension (at approximately 1 × 10^9^ CFU/mL) cultivated overnight in MRS broth was spotted on the surface of MRS agar containing 1.5% (*w*/*v*) agar and incubated for 24 h at 37°C. A 100 *μ*L aliquot of *G*. *vaginalis* or *E*. *coli* suspension was then mixed with 100 mL of soft BHIS agar or LB agar (0.7% agar) (final viable count of approximately 1 × 10^6^ CFU/mL) and poured over the spot-inoculated MRS agar. The plates were incubated aerobically at 37°C for 48 h. The antagonistic activity was determined by the diameter (mm) of growth inhibition zones around each spot, corrected for the diameter of the spot. Uninoculated MRS agar was used as a negative control.

Generally, the antibacterial constituents produced by *Lactibacillus* were hydrogen peroxide, organic acids, and bacteriocins [24]. In order to examine the antibacterial mechanism, a well diffusion test was used as previously described [13]. The cultures were centrifuged (8000 × *g*, 20 min) and sterile filtered (0.22 *μ*m). The supernatants were treated with catalase (300 IU/mL, 37°C, 1 h, Macklin, China) and 1 M NaOH (final pH 6.5), in order to eliminate the effects of organic acid and hydrogen peroxide. These cell-free neutralized supernatants (CFN) were used as a bacteriocin solution and stored for further use. One hundred microliter aliquots of CFN were added into the 6 mm diameter holes on each agar plate. MRS broth was added into holes of the control group. After 2 h, the liquid was volatilized, and the bacteriocins were permeated into the agar; then, 100 *μ*L of *G*. *vaginalis* (1 × 10^6^ CFU/mL) was spread evenly on the surface of the agar plates. Plates were incubated at 37°C for 48 h, and the diameters of the inhibition growth zones were measured. All experiments were performed in triplicate.

### 2.4. Antibacterial Testing of Untreated Cell-Free Supernatant (CFS) and CFN

To determine the antibacterial activity of the CFS and CFN, *G*. *vaginalis* was grown at 37°C for 24 h in BHIS broth. The *G*. *vaginalis* culture was diluted with BHIS broth medium by 5% to 5 × 10^7^ CFU/mL. Then, 100 *μ*L of *G*. *vaginalis* suspension and 100 *μ*L of CFS and CFN were added to the wells of a 96-well microtiter plate in five replicates for each *Lactobacillus* CFS and CFN. One hundred microliters of BHIS broth and 100 *μ*L of CFS and CFN were added to the wells of a 96-well microtiter plate in three replicates for each *Lactobacillus* CFS and CFN as blanks. The plates were then incubated anaerobically at 37°C for 24 h. In the control wells, the CFS or CFN was replaced by sterile MRS broth. The optical density (OD) at 595 nm was recorded after incubation using a microplate reader (Thermo Scientific 3001, USA). The growth was calculated as the measured value minus the average blank value.

### 2.5. Antibiotic Susceptibility Testing

The minimum inhibitory concentrations (MIC) of cefoxitin sodium, ampicillin, kanamycin, gentamycin, erythromycin, tetracycline, polymyxin B, chloramphenicol, nalidixic acid, linezolid, metronidazole, and clindamycin against the selected *Lactobacilli* strains were determined using a broth microdilution test as previously described [25]. The plates were statically incubated at 37°C for 24 h. Subsequently, the bacterial growth was visually observed, and the minimum inhibitory concentration of each antibiotic was confirmed as the lowest concentration capable of inhibiting visible bacterial growth.

### 2.6. Detection of the Plantaricin-Related Genes

The presence of plantaricin-related genes was investigated in five *Lactobacillus* isolates by PCR with specific primers, as previously described [26]. In addition, the *entA*, *gasA*, and *laf* genes were also detected using primers as previously reported [27–29]. All primers were designed based on published sequences and chemically synthesized by Invitrogen Biotechnology Company (Shanghai, China) (Supplementary Table S1). The PCR products were sent to Invitrogen Biotechnology Company (Shanghai, China) for sequencing (Supplementary Figure S1-S16). The obtained sequences were compared with known sequences in the NCBI database using the MegAlign software 8(DNASTAR, Inc., Madison, USA). If the similarity index was more than 90%, the strain was positive for bacteriocin. If the similarity index was less than 90% or there were no PCR products, the strain was considered bacteriocin negative.

### 2.7. Virulence Genes in G. vaginalis Affected by Lactobacillus

To investigate interactions between *Lactobacillus* and *G*. *vaginalis* in Hela cell monolayers, we used a medium simulating genital tract secretions [8]. We studied two conditions for this. First, Hela cells were covered with *Lactobacillus* before infecting them with *G*. *vaginalis* (*G*. *vaginalis* infection in Hela with Lac). Second, *G*. *vaginalis* was allowed to adhere to Hela cells and then *Lactobacillus* was added (*G. vaginalis* infection in Hela treated with Lac). For the first condition, a *Lactobacillus* suspension, adjusted to 1 × 10^9^ CFU/mL, was added to a monolayer of Hela cells for 3 h. Afterwards, the Hela cells were washed with PBS twice. Then, a *G*. *vaginalis* suspension, adjusted to the same concentration, was added to Hela cell monolayers pretreated with *Lactobacillus* for 3 h. Next, the monolayer was washed with PBS three times. The *G*. *vaginalis* that adhered to Hela cells after 3 h served as a control. For the second condition, a *G*. *vaginalis* suspension was added to a monolayer of Hela cells for 3 h and then washed twice with PBS. The *Lactobacillus* suspension was then added to the Hela cells infected with *G. vaginalis* for 3 h and then washed with PBS three times. The *G. vaginalis* that adhered to Hela cells after 3 h served as a control. Total RNA from these two conditions was extracted using TRIzol (Beyotime, China) according to the manufacturer's instructions. RNA concentration and purity were determined with a Microplate Reader (BioTek, USA). Finally, the ReverTra Ace qPCR RT Kit (TOYOBO, Japan) was used to reverse transcribe 2 *μ*g of total RNA into cDNA.

Using qPCR, we examined the effect of *Lactobacillus* on the expression levels of three target genes (*HMPREF0424_1122*, *HMPREF0424_0156*, and *HMPREF0424_0354*) involved in antimicrobial resistance in *G*. *vaginalis* [30]. The cDNA was amplified by real-time quantitative PCR using SYBR Green Realtime PCR Master Mix (TOYOBO, Japan) on a StepOnePlus system (Applied Biosystems, USA) with primers listed in Supplementary Table S1. The real-time PCR conditions were as follows: 95°C for 1 min, followed by 40 cycles of denaturation at 95°C for 15 s, annealing at 60°C for 15 s, and extension and fluorescent data collection at 72°C for 45 s. For melting curve analysis, the temperature was decreased from 95°C to 65°C at a rate of 0.1°C/s with continuous acquisition of the fluorescence signal intensity. Data were analyzed using Applied Biosystems software, and differences in mRNA expression levels were calculated after normalizing to the 16S rRNA level. Results are expressed as the fold change relative to that in the control group based on ^*ΔΔ*Ct^ value analysis [31].

### 2.8. Statistical Analysis

Experimental results were analyzed for statistical significance using GraphPad Prism (GraphPad, San Diego, USA). Independent Student *t*-test analysis was performed. The statistical significance level was defined as *P* < 0.05. All the results were expressed as the mean ± SD.

## 3. Results

### 3.1. Isolation and Identification of Lactobacillus Strains

**Figure 1:**
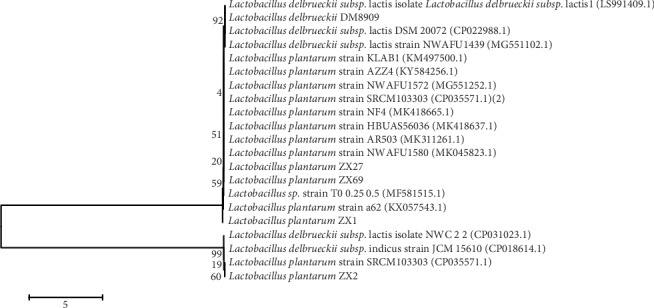
Phylogenetic analysis of strains of ZX1, ZX2, ZX27 ZX69, and DM8909 based on 16S rDNA partial gene sequences. The remaining sequences of the *Lactobacillus* in this figure were downloaded from NCBI.

One hundred and five bacillus-shaped and Gram stain positive colonies were selected from ten yogurt samples and were then authenticated by 16S rDNA gene sequencing. The 37 strains identified as *Lactobacillus* were selected for antibacterial tests against *G*. *vaginalis in vitro* by agar spot testing. Finally, four strains named ZX1, ZX2, ZX27, and ZX69 were selected for further study. Phylogenetic analysis of ZX1, ZX2, ZX27, and ZX69 showed high homology with *L*. *plantarum* (Figure 1).

### 3.2. Antibacterial Properties Determined In Vitro by Agar Spot and Well Diffusion Tests

The four new *Lactobacillus s*trains isolated from old yogurts and a commercial strain DM8909 were screened for their antimicrobial activity by agar spot and well diffusion tests (Table 1). In the agar spot assay, the growth inhibition zone diameters with ZX1, ZX2, ZX27, and ZX69 were ≥10 mm for *G*. *vaginalis* and ≥20 mm for *E*. *coli*. The zones with DM8909 were 3.64 mm for *G*. *vaginalis* and 14.50 mm for *E*. *coli*, which were much lower values than those obtained with the four newly isolated *Lactobacillus* strains. Similarly, in a well diffusion test, the CFN of the four isolated *Lactobacillus* strains had inhibitory activity against *G*. *vaginalis* with growth inhibition zone diameters varying from 1.46 to 3.02 mm. The CFN of DM8909 did not inhibit the growth of *G*. *vaginalis.* The most robust antagonistic activities for *G. vaginalis* in the agar spot and well diffusion assays were displayed by *L*. *plantarum* ZX27.

### 3.3. Antibacterial Testing of CFS and CFN against G. vaginalis

**Figure 2:**
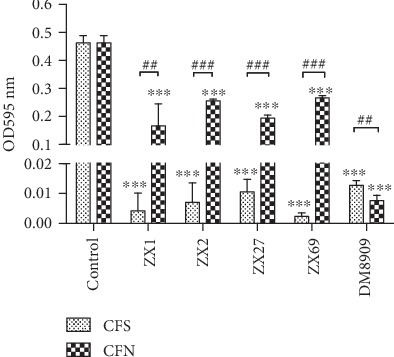
*Gardnerella vaginalis* growth in the presence of untreated cell-free supernatant (CFS) and cell-free neutralized supernatant (CFN). Control: *G. vaginalis* growth in BHIS broth added in 100 *μ*L sterile MRS broth. ^∗∗∗^*P* < 0.001 compared with *G. vaginalis* growth in BHIS broth as the control.

The CFS and CFN of the five *Lactobacillis* showed strong significant inhibitory effects (Figure 2) on the growth of *G*. *vaginalis* (*P* < 0.001). There was no significant difference in the potency of the inhibitory effect between the CFS from each of the five samples (*P* > 0.05). However, after neutralizing the supernatant acidity and hydrogen peroxide, the antimicrobial effect was significantly reduced (*P* < 0.01) compared with the respective CFS except for DM8909, which still showed a significant inhibition (*P* < 0.001) of *G*. *vaginalis* growth. The antimicrobial effect of CFN of DM8909 was significantly enhanced (*P* < 0.01) compared with the CFS, which was opposite to other four tested strains, indicating that acidity and hydrogen peroxide were not the main antimicrobial elements of the CFS from this strain against *G*. *vaginalis*.

### 3.4. Antibiotic Resistance

The studied *Lactobacilli* strains did not show resistance to erythromycin, linezolid, or clindamycin. Four out of the five strains were resistant to kanamycin and tetracycline (*L*. *plantarum* ZX1, ZX2, ZX27, and ZX69) and DM8909 was sensitive to kanamycin and tetracycline. Two out of the five strains were resistant to ampicillin (*L*. *plantarum* ZX27, ZX69) and chloramphenicol (*L*. *plantarum* ZX2, ZX27). All of the strains were resistant to cefoxitin sodium, gentamicin, polymyxin B, nalidixic acid, and metronidazole (Table 2). Overall, the resistance profiles to antibiotics varied among the *Lactobacillus* strains. Resistance was determined according to the cut-offs recommended by the European Food Safety Authority (EFSA, 2012) [25].

### 3.5. Sequencing of the Bacteriocin Gene

In an attempt to determine whether the selected strains carried genes for the production of known plantaricins and other common bacteriocins, PCR analysis using primers specific for individual bacteriocin genes was used. All five strains were tested positive for *plnA*, *plnB*, *plnC*, *plnD*, *plnEF*, *plnI*, *plnJ*, *plnK*, *plnG*, and *plnN*, suggesting that they could produce all the plantaricin peptides described in strain C11 [32]. None of the strains were positive for the plantaricins NC8, S, and W. In addition, none of the strains were positive for genes encoding *entA*, *gasA*, and *laf*, which are genes that are frequently found in *Enterococcus faecalis*, *Lactobacillus casei*, and/or lactic acid bacteria (Table 3).

### 3.6. Virulence Genes in G. vaginalis Are Affected by Lactobacillus

**Figure 3:**
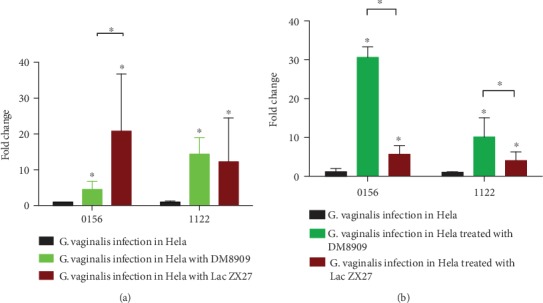
Alterations in gene expression profiles associated with exposure of *Lactobacillus*, in (a) *G. vaginalis* infection in Hela with Lac and (b) *G. vaginalis* infection in Hela treated with Lac in each panel; fold change refers to the mean levels of gene expression across replicates calculated using the ^*ΔΔ*Ct^ method relative to levels in the untreated control. Fold change = 2^−ΔΔCt^. Asterisks indicate statistically significant differences in the expression of each gene between treated samples and controls, as analyzed by one-way ANOVA with Dunnett's posttesting for multiple testing (^∗^*P* ≤ 0.05). Error bars indicate standard deviation.

We used qPCR to evaluate and compare the effects on *G*. *vaginalis* cells after exposure to two *Lactobacillus* strains for 3 h. Expression levels of three genes previously shown to be involved in antimicrobial resistance in *G*. *vaginalis* were compared to those in control untreated cells prepared under the same conditions without *Lactobacillus*. Among the three genes, *HMPREF0424_0354* was not detected. Regardless of the conditions of *Lactobacillus* treatment, significant increases in the expression of *HMPREF0424_0156* and *HMPREF0424_1122* were observed in adherent *G*. *vaginalis* cells in the presence of *Lactobacillus*. Further, the effects of different *Lactobacillus* strains were variable for different genes. When *G*. *vaginalis* infected Hela cells pretreated with *Lactobacillus*, *HMPREF0424_0156*, which encodes bacitracin transport ATP-binding protein *BcrA*, was significantly upregulated by DM8909 (fourfold) and by ZX27 (20-fold) (Figure 3(a)). *HMPREF0424_1122*, which encodes a multidrug resistance ABC transporter, was also significantly upregulated by DM8909 (14-fold) and by ZX27 (13-fold). When *G*. *vaginalis* had already adhered to Hela cells, *Lactobacillus* also upregulated *HMPREF0424_0156* by 30-fold (DM8909) and 5-fold (ZX27) (Figure 3(b)). Meanwhile, *HMPREF0424_1122* was increased by 10-fold (DM8909) and four-fold (ZX27).

## 4. Discussion

BV is one of the most common diseases in women of child-bearing age and is typically associated with the presence of the pathogenic bacteria, *Gardnerella vaginalis*. The female genital tract is dominated by *Lactobacillus* spp. in approximately 70% of women [5]. *Lactobacillus* species play an essential role in maintaining the ecosystem of the vagina [33–35]. *Lactobacillus* has proven to be efficient in treating BV by producing organic acids, hydrogen peroxide, bacteriocins, and adhesion inhibitors [34].

The *Lactobacillus* sp. used in this study was *L*. *plantarum* ZX1, ZX2, ZX27, and ZX69 and *L*. *delbrueckii* DM8909 (control strain). Four strains (*L*. *plantarum* ZX1, ZX2, ZX27, and ZX69) were isolated from yogurt samples and were identified as *L*. *plantarum* based on 16S sequencing. The five tested *Lactobacillus* strains displayed the ability to inhibit the pathogenic bacteria *G*. *vaginalis* and *E*. *coli*. The antibacterial effects of *L*. *delbrueckii* DM8909 were weaker than the four new isolated strains in both agar spot and well diffusion tests, determined by the size of the zone of inhibition. In terms of DM8909, the inhibition diameter of CFN was <1 mm and the inhibition diameter in the agar spot test was 3.67 mm, both of which were much lower than the diameters of inhibition created by the four isolated *Lactobacillus* strains. Our findings were consistent with Wang et al.'s research [36], who reported that *L*. *plantarum* isolated from Tibetan yaks could strongly inhibit the growth of *E*. *coli* and *S*. *aureus*. Andreeva et al. [37] isolated a *Lactobacillus* sp. strain VLb3 from healthy Bulgarian women, which had a diameter of 12 ± 1 mm in inhibition against *G*. *vaginalis* ATCC14018 by its CFS and CFN using well diffusion tests. In a study [38], three strains of *Lactobacillus* isolated from cocoa fermentation had an activity against *G*. *vaginalis* ATCC 49154 using the agar diffusion technique and the supernatant halos of *L*. *plantarum* 6.2 and *L*. *plantarum* 7.1 were 12 and 11 mm, respectively. Another research showed that the CFS of some LAB had an inhibitory effect on the growth of *G*. *vaginalis* BCRC 17040 (inhibition diameter was about 2-3 mm) [39]. In above three research, the inhibition zone against *G*. *vaginalis* was about 3 mm after subtracting the well diameter, and the result in our study was 1.46 to 3.02 mm. The results were abnormal for *E*. *coli*. The CFN of ZX1, ZX27, and DM8909 had no bacteriostatic activity. We thus speculated that the antibacterial properties of CFN from *Lactobacillus* have different antibacterial spectra.

The tested *Lactobacillus* strains caused a significant reduction in the microbial growth of *G*. *vaginalis* in BHIS broth, as determined by a change in the OD at 595 nm. Jeong et al. also found that *Lactobacillus kefiranofaciens* DD2, isolated from kefir, can inhibit the growth of *Sreptococcus mutans* and *Sreptococcus sobrinus* using the same methods [31]. At the same time, there was no significant difference between the CFS from the different *Lactobacillus* species regardless of their metabolic pattern. The facultative heterofermentative organism *L. plantarum* and the strictly homofermentative organism *L*. *delbrueckii* had similar antimicrobial effects (Figure 2). To determine the effect of bacteriocins produced by the tested *Lactobacillus* strains, the CFS was treated with NaOH and catalase to neutralize the pH and hydrogen peroxide [13, 23]. It was observed that the neutralized CFS (CFN) caused a lower reduction in microbial growth than CFS, but CFN still showed a significant reduction in *G*. *vaginalis* growth when compared to the control. Wasfi et al. found that when CFS was treated to pH = 7, the ability of all 4 *Lactobacillus* inhibiting the growth of *Streptococcus mutans* is significantly declining (*P* < 0.01) [40]. Our results of the four *L. plantarum* are consistent with the results. DM8909 CFN had more significant growth inhibition than the CFS, suggesting that the main antimicrobial elements produced by DM8909 against *G*. *vaginalis* were bacteriocins. We assumed that the bacteriocins of DM8909 contribute to its antimicrobial effect at pH 6.5. Organic acids or hydrogen peroxide produced by the four new *L*. *plantarum* isolates ZX1, ZX2, ZX27, and ZX69 have antimicrobial effects on *G*. *vaginalis*. In the well diffusion assay, the inhibition diameter of DM8909 was less than 1 mm, indicating that the CFN of DM8909 had no inhibition effects. Nevertheless, 100 *μ*L of this CFN inhibited the growth of *G*. *vaginalis* directly. One possibility for this discrepancy is that the inhibitory component of the DM8909 CFN could not diffuse in the agar plate.

For the four strains to be considered potential probiotics, they must be safe for human consumption. *L*. *plantarum* ZX27 and ZX69 were resistant to ampicillin, and *L*. *plantarum* ZX2 and ZX27 were resistant to chloramphenicol. All of the strains were resistant to cefoxitin sodium, gentamicin, polymyxin B, nalidixic acid, and metronidazole. The nature of this resistance warrants further studies before any of the strains can be considered safe for human use. In another study, three *L*. *fermentum*, which have anti-*G*. *vaginalis* activity, were sensitive to ampicillin and chloramphenicol [38]. Generally, *Lactobacillus* are safe probiotics, but some safety tests should be performed before using *Lactobacillus* in a clinical setting. In our study, the five *Lactobacillus* strains were resistant to the tested antibiotics.

All the CFN of the five strains have antimicrobial activities against *G*. *vaginalis*, indicating that the five strains can produce bacteriocins. Interestingly, the five stains in our study were positive for the genes *plnA*, *plnB*, *plnC*, *plnD*, *plnEF*, *plnI*, *plnJ*, *plnK*, *plnG*, and *plnN*, but the inhibitory effects of different strains were different between the five strains. The phenomenon suggested that the antibacterial effects were strain specific. This may be because the gene expression patterns or bacteriocin secretion between these strains are different in the same culture conditions. Omar et al. isolated *L. plantarum* from ben saalga; however, they found only one strain (5.2.2) that possessed all the plantaricin genes, besides the *plnB* gene [26].This is the first report that *L*. *delbrueckii* DM8909 has plantaricin genes. *L*. *delbrueckii* is the subspecies of *Lactobacillus bulgaricus* and is used in dairy fermentation broadly. It was unusual that *L*. *bulgaricus* possessed the *pln* locus responsible for bacteriocin biosynthesis in *L*. *plantarum* C11, because the *pln* locus encoded plantaricins in *L*. *plantarum* [17, 32]. The reported *Lactobacillus* which have been found to harbor *pln* genes were all *L*. *plantarum* (C11, NC8, WCFS1, J23, and J51) [41].The result was consistent with the phylogenetic analysis in which the *L*. *delbrueckii* DM8909 was close to *L*. *plantarum* in the evolutionary system. Meanwhile, the CFN of DM8909, which contains bacteriocin, can inhibit *G*. *vaginalis* growth in BHIS broth directly.

Using an *in vitro* model, we tested gene expression in *G*. *vaginalis* after exposure to *Lactobacillus*. Remarkably, precoating the Hela monolayer with *Lactobacillus* or treating the cells with *Lactobacillus* after *G*. *vaginalis* had already adhered and enhanced the expression of the *HMPREF0424_0156* and *HMPREF0424_1122* transcripts. However, the degree of upregulation with the two *Lactobacillus* strains was different. In the precoating model, DM8909 upregulated *HMPREF0424_0156* transcripts fourfold, whereas ZX27 increased the expression by 20-fold (*P* < 0.05). In the second (treatment) model, DM8909 upregulated *HMPREF0424_0156* and *HMPREF0424_1122* to a greater extent than ZX27 (*P* < 0.05). This result reminds us that *Lactobacillus* will affect the gene expression in *G. vaginalis* and that different strains will have diverse regulatory effects.

## 5. Conclusion

The results of this study showed that the four new *L*. *plantarum* isolates could inhibit the growth of *G*. *vaginalis*, a causative agent of BV. The four new *L*. *plantarum* isolates and the commercial strain *L. delbrueckii* DM8909 were positive for genes related to plantaricins, which have antimicrobial activity. Our findings support that these *L*. *plantarum* strains could be used as probiotics for treating the BV disease [42, 43]. *Lactobacillus* can upregulate the transcription levels of antimicrobial resistance genes in *G. vaginalis.* We speculated that the possible mechanism underlying this phenomenon is that bacteriocins produced by *Lactobacillus* induce altered gene transcription in *G*. *vaginalis*. Further studies, including survival rate in vaginal conditions and adhesion assay to VK2/E6E7 or Hela cells, should be conducted, as well as *in vivo* studies, to verify the potential health benefits of the new *L*. *plantarum* strains.

## Figures and Tables

**Table 1:** Antimicrobial activity of *Lactobacillus* isolates determined by agar spot and well diffusion tests.

Strain	*G. vaginalis* ATCC49145		*E. coli* ATCC25922	
	Spot ager	Well diffusion of CFN	Spot ager	Well diffusion of CFN
ZX1	10.67 ± 0.27	2.94 ± 0.32	22.37 ± 1.65	<1(±0.00)
ZX2	10.00 ± 1.41	1.56 ± 0.13	25.83 ± 1.66	1.38 ± 0.1
ZX27	13.67 ± 1.70	3.02 ± 0.43	27.77 ± 2.49	<1(±0.00)
ZX69	10.67 ± 0.94	1.46 ± 0.22	20.77 ± 0.56	2.52 ± 0.65
DM8909	3.67 ± 0.47	<1(±0.00)	14.50 ± 1.50	<1(±0.00)

**Table 2:** MIC of different antibiotics against the *Lactobacillus* stains.

Antibiotics	Strains				
	ZX1	ZX2	ZX27	ZX69	DM8909
Cefoxitin sodium	>512	>512	>512	>512	>512
Ampicillin	32	32	64	128	1
Kanamycin	>512	>512	>512	>512	32
Gentamycin	>512	512	>512	>512	512
Erythromycin	0.25	1	0.5	<0.25	<0.25
Tetracycline	256	256	512	128-256	4-8
Polymyxin B	>512	512	>512	512	128
Chloramphenicol	16	64	64	16-32	8
Nalidixic acid	>512	>512	>512	>512	>512
Linezolid	4	8	16	32-64	4
Metronidazole	>512	>512	>512	>512	>512
Clindamycin	0.5	0.5	<0.25	<0.25	0.5

**Table 3:** PCR-based detection of plantaricin genes and other bacteriocinogenic genes in *L. plantarum* strains and *L. delbrueckii* DM8909.

Bacteriocinogenic genes	Strains				
	ZX1	ZX2	ZX27	ZX69	DM8909
*plnA*	+	+	+	+	+
*plnB*	+	+	+	+	+
*plnC*	+	+	+	+	+
*plnD*	+	+	+	+	+
*plnEF*	+	+	+	+	+
*plnI*	+	+	+	+	+
*plnJ*	+	+	+	+	+
*plnK*	+	+	+	+	+
*plnG*	+	+	+	+	+
*plnN*	+	+	+	+	+
Plantaricin NC8 structural gene	-	-	-	-	-
Plantaricin S structural gene	-	-	-	-	-
Plantaricin W structural gene	-	-	-	-	-
*entA*	-	-	-	-	-
*gasA*	-	-	-	-	-
*laf*	-	-	-	-	-

## Data Availability

If requested, the original data of this article is available.

## Additional Points

*Research Involving Human Participants and/or Animals*. This article does not contain any studies on either animals or human participants.

## Supplementary Materials

Supplementary Materials Primer sequences used for PCR or RT-qPCR assay in the article and the gene electrophoretogram and sequences of bacteriocin.

## References

[1] Primary C., Sexual C., Workstream H. (2007). Bacterial vaginosis. *Medicine*.

[2] Falagas M. E., Betsi G. I., Athanasiou S. (2007). Probiotics for the treatment of women with bacterial vaginosis. *Clinical Microbiology and Infection*.

[3] Palmeira-de-Oliveira R., Palmeira-de-Oliveira A., Martinez-de-Oliveira J. (2015). New strategies for local treatment of vaginal infections. *Advanced Drug Delivery Reviews*.

[4] Servin A. L. (2004). Antagonistic activities of *Lactobacilli* and bifidobacteria against microbial pathogens. *FEMS Microbiology Reviews*.

[5] Petrova M. I., Lievens E., Malik S., Imholz N., Lebeer S. (2015). *Lactobacillus* species as biomarkers and agents that can promote various aspects of vaginal health. *Frontiers in Physiology*.

[6] Walker A. W. (2016). Studying the Human Microbiota. *Microbiota of the Human Body*.

[7] Reid G. (2017). The development of probiotics for women’s health. *Canadian Journal of Microbiology*.

[8] Castro J., Martins A. P., Rodrigues M. E., Cerca N. (2018). *Lactobacillus* crispatus represses vaginolysin expression by BV associated *Gardnerella vaginalis* and reduces cell cytotoxicity. *Anaerobe*.

[9] Castro J., Alves P., Sousa C. (2015). Using an _in-vitro_ biofilm model to assess the virulence potential of *Bacterial Vaginosis* or non-*Bacterial Vaginosis* _Gardnerella vaginalis_ isolates. *Scientific Reports*.

[10] Jang S.-E., Jeong J.-J., Choi S.-Y., Kim H., Han M., Kim D.-H. (2017). *Lactobacillus rhamnosus* HN001 and *Lactobacillus acidophilus* La-14 attenuate *Gardnerella vaginalis*-infected bacterial vaginosis in mice. *Nutrients*.

[11] Joo H. M., Hyun Y. J., Myoung K. S. (2011). *Lactobacillus johnsonii* HY7042 ameliorates *Gardnerella vaginalis*-induced vaginosis by killing *Gardnerella vaginalis* and inhibiting NF-*κ*B activation. *International Immunopharmacology*.

[12] Shamshu R., Vaman J., Nirmala C. (2017). Role of probiotics in lower reproductive tract infection in women of age group 18 to 45 years. *International Journal of Reproduction, Contraception, Obstetrics and Gynecology*.

[13] Ołdak A., Zielińska D., Rzepkowska A., Kołozyn-Krajewska D. (2017). Comparison of antibacterial activity of *Lactobacillus plantarum* strains isolated from two different kinds of regional cheeses from Poland: oscypek and korycinski cheese. *BioMed Research International*.

[14] Berta G., Chebeñová V., Brežná B., Pangallq D., Valík L., Kuchta T. (2009). Identification of lactic acid bacteria in Slovakian bryndza cheese. *Journal of Food & Nutrition Research*.

[15] Devi S. M., Kurrey N. K., Halami P. M. (2018). In vitro anti-inflammatory activity among probiotic *Lactobacillus* species isolated from fermented foods. *Journal of Functional Foods*.

[16] Mandracchia V. J., Hayes D. W., Yoho R. M., Hayes M. F. (2000). Diagnosis, differential and treatment options. *Nature Reviews Microbiology*.

[17] Kaškonienė V., Stankevičius M., Bimbiraitė-Survilienė K. (2017). Current state of purification, isolation and analysis of bacteriocins produced by lactic acid bacteria. *Applied Microbiology and Biotechnology*.

[18] Cotter P. D., Hill C., Ross R. P. (2005). Bacteriocins: developing innate immunity for food. *Nature Reviews Microbiology*.

[19] Yin X., Heeney D., Srisengfa Y., Golomb B., Griffey S., Marco M. (2018). Bacteriocin biosynthesis contributes to the anti-inflammatory capacities of probiotic *Lactobacillus plantarum*. *Beneficial Microbes*.

[20] McFarland L. V. (2015). From yaks to yogurt: the history, development, and current use of probiotics. *Clinical Infectious Diseases*.

[21] Zhu Y., Xiao L., Shen D., Hao Y. (2010). Competition between yogurt probiotics and periodontal pathogens in vitro. *Acta Odontologica Scandinavica*.

[22] Pu F., Guo Y., Li M. (2017). Yogurt supplemented with probiotics can protect the healthy elderly from respiratory infections: a randomized controlled open-label trial. *Clinical Interventions in Aging*.

[23] Garcia E. F., Luciano W. A., Xavier D. E. (2016). Identification of lactic acid bacteria in fruit pulp processing byproducts and potential probiotic properties of selected *Lactobacillus* strains. *Frontiers in Microbiology*.

[24] Vahedi Shahandashti R., Kasra Kermanshahi R., Ghadam P. (2016). The inhibitory effect of bacteriocin produced by *Lactobacillus* acidophilusATCC 4356 and *Lactobacillus plantarum* ATCC 8014 on planktonic cells and biofilms of Serratia marcescens. *Turkish Journal of Medical Sciences*.

[25] EFSA Panel on Additives and Products or Substances used in Animal Feed (FEEDAP) (2012). Guidance on the assessment of bacterial susceptibility to antimicrobials of human and veterinary importance. *EFSA Journal*.

[26] Omar N. B., Abriouel H., Lucas R., Martínez-Cañamero M., Guyot J.-P., Gálvez A. (2006). Isolation of bacteriocinogenic *Lactobacillus plantarum* strains from ben saalga, a traditional fermented gruel from Burkina Faso. *International Journal of Food Microbiology*.

[27] Du Toit M., Franz C. M. A. P., Dicks L. M. T., Holzapfel W. H. (2000). Preliminary characterization of bacteriocins produced by Enterococcus faecium and Enterococcus faecalis isolated from pig faeces. *Journal of Applied Microbiology*.

[28] Stoyancheva G., Marzotto M., Dellaglio F., Torriani S. (2014). Bacteriocin production and gene sequencing analysis from vaginal *Lactobacillus* strains. *Archives of Microbiology*.

[29] Macwana S. J., Muriana P. M. (2012). A ‘bacteriocin PCR array’ for identification of bacteriocin-related structural genes in lactic acid bacteria. *Journal of Microbiological Methods*.

[30] Castro J., França A., Bradwell K. R., Serrano M. G., Jefferson K. K., Cerca N. (2017). Comparative transcriptomic analysis of *Gardnerella vaginalis* biofilms vs. planktonic cultures using RNA-seq. *NPJ Biofilms Microbiomes*.

[31] Jeong D., Kim D. H., Song K. Y., Seo K. H. (2018). Antimicrobial and anti-biofilm activities ofLactobacillus kefiranofaciensDD2 against oral pathogens. *Journal of Oral Microbiology*.

[32] Diep D. B., Håvarstein L. S., Nes I. F. (1996). Characterization of the locus responsible for the bacteriocin production in *Lactobacillus plantarum* C11. *Journal of Bacteriology*.

[33] Amabebe E., Anumba D. O. C. (2018). The vaginal microenvironment: the physiologic role of *Lactobacill*i. *Frontiers in Medicine*.

[34] Tachedjian G., Aldunate M., Bradshaw C. S., Cone R. A. (2017). The role of lactic acid production by probiotic *Lactobacillus* species in vaginal health. *Research in Microbiology*.

[35] Borges S., Silva J., Teixeira P. (2014). The role of *Lactobacilli* and probiotics in maintaining vaginal health. *Archives of Gynecology and Obstetrics*.

[36] Wang L., Zhang H., Rehman M. U. (2018). Antibacterial activity of *Lactobacillus plantarum* isolated from Tibetan yaks. *Microbial Pathogenesis*.

[37] Patil P. S. (2016). International journal of advanced research in biological sciences. *International Journal of Advanced Research in Biological Sciences*.

[38] Pessoa W. F. B., Melgaço A. C. C., de Almeida M. E., Ramos L. P., Rezende R. P., Romano C. C. (2017). *In vitro* activity of *Lactobacilli* with probiotic potential isolated from cocoa fermentation against *Gardnerella vaginalis*. *BioMed Research International*.

[39] Tsai C. C., Lai T. M., Hsieh Y. M. (2019). Evaluation of *Lactobacilli* for antagonistic activity against the growth, adhesion and invasion of *Klebsiella pneumoniae* and *Gardnerella vaginalis*. *Indian Journal of Microbiology*.

[40] Wasfi R., Abd El-Rahman O. A., Zafer M. M., Ashour H. M. (2018). Probiotic *Lactobacillus sp.* inhibit growth, biofilm formation and gene expression of caries-inducing Streptococcus mutans. *Journal of Cellular and Molecular Medicine*.

[41] Diep D. B., Straume D., Kjos M., Torres C., Nes I. F. (2009). An overview of the mosaic bacteriocin pln loci from *Lactobacillus plantarum*. *Peptides*.

[42] Mastromarino P., Macchia S., Meggiorini L. (2009). Effectiveness of *Lactobacillus*-containing vaginal tablets in the treatment of symptomatic bacterial vaginosis. *Clinical Microbiology and Infection*.

[43] Heczko P. B., Tomusiak A., Adamski P. (2015). Supplementation of standard antibiotic therapy with oral probiotics for bacterial vaginosis and aerobic vaginitis: a randomised, double-blind, placebo-controlled trial. *BMC Women's Health*.

