# Supplementary material for: Antibacterial Activity of Lactobacillus Strains Isolated from Mongolian Yogurt against Gardnerella vaginalis
Source: Biomed Res Int. 2020 Apr 22;2020:3548618. doi: 10.1155/2020/3548618 (PMC7195648; doi:10.1155/2020/3548618)
Supplement: Supplementary Materials — Primer sequences used for PCR or RT-qPCR assay in the article and the gene electrophoretogram and sequences of bacteriocin. [file 3548618.f1.pdf]

Supplementary material

Supplementary Table S1: Primer sequences used for PCR or RT-qPCR assay

Target	PCR primers	Annealing temp. (°C)	Amplicon size (bp)	Ref.
plnA	F: GTA CAG TAC TAA TGG GAG	53	450	14
	R: CTT ACG CCA ATC TAT ACG			
plnB	F: TTC AGA GCA AGC CTA AAT GAC	51.5	165	14
	R: GCC ACT GTA ACA CCA TGA C			
plnC	F:AGC AGA TGA AAT TCG GCA G	49.5	108	14
	R: ATA ATC CAA CGG TGC AAT CC			
plnD	F: TGA GGA CAA ACA GAC TGG AC	53	414	14
	R: GCA TCG GAA AAA TTG CGG ATA C			
plnEF	F: GGC ATA GTT AAA ATT CCC CCC	53.2	428	14
	R:CAG GTT GCC GCA AAA AAA G			
plnI	F: CTC GAC GGT GAA ATT AGG TGT AAG	52.5	450	14
	R: CGT TTA TCC TAT CCT CTA AGC ATT GG			
plnJ	F: TAA CGA CGG ATT GCT CTG	51	475	14
	R: AAT CAA GGA ATT ATC ACA TTA GTC			
plnK	F:CTG TAA GCA TTG CTA ACC AAT C	52.9	246	14
	R: ACT GCT GAC GCT GAA AAG			
plnG	F:TGC GGT TAT CAG TAT GTC AAA G	52.8	453	14
	R: CCT CGA AAC AAT TTC CCC C			
plnN	F:ATT GCC GGG TTA GGT ATC G	51.9	146	14
	R: CCT AAA CCA TGC CAT GCA C			
Plantaricin NC8 structural gene	F: GGT CTG CGT ATA AGC ATC GC	60	207	14
	R: AAA TTG AAC ATA TGG GTG CTT TAA ATT CC			
Plantaricin S structural gene	F: GCC TTA CCA GCG TAA TGC CC	60	320	14
	R: CTG GTG ATG CAA TCG TTA GTT T			
Plantaricin W structural gene	F: TCA CAC GAA ATA TTC CA	55	165	14
	R: GGC AAG CGT AAG AAA TAA ATG AG			
entA	F:AAA TAT TAT GGA GTG TAT	56	159	15
	R:GCA CTT CCC TGG AAT TGC TC			
gasA	F: GAA CAG GTG CAC TAA TCG GT	62	800	16
	R: CAG CTA AGT TAG AAG GGG CT			
laf	F:AGT CGT TGT TGG TGG AAG AAA T	62	184	17
	R:TCT TAT CTT GCC AAA ACC ACC T			
HMPREF0424_1122	F: CAGCACCTGTAGCTCCAACA	60	195	19
	R:TGGCTCAAGAGATTGTGTGC			
HMPREF0424_0156	F: CCGACCGCATACCTATTTTG	60	178	19
	R: GCAAGACGGTCTCCAAACTC			
HMPREF0424_0354	F: AACCAAGCAATTCCACAAGC	60	199	19
	R: CCGTCGTTTTGGCAGTATTT			
16S RNA	F: TGAGTAATGCGTGACCAACC	60	167	19
	R: AGCCTAGGTGGGCCATTACC			

The primers of *plnA* to *laf* were used to detect the the plantaricin-related genes in the five newly isolated strains. The primers *HMPREF0424_1122* to *16S RNA* were used to detect the virulence genes in *G. vaginalis*.

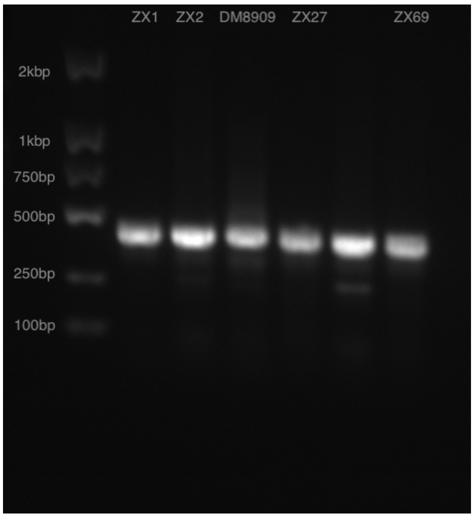

Supplementary Figure S1: The p*lnA* gene detection results of 5 strains.

The sequencing results of the *plnA*(450bp) are the following:

nAsTnttdSsTCTTACGCCCATCTATACGAAATATAACTTGAAAAACAGTTATT
CAATAAATCAATTACCATCCCCATTTTTTAAACAGTTTCTTTACCTGTTTAA
TTGCAGTTGCCCCCATCTGCAAAGAATACGCACTACTCTTTCCACCTACTA
TTTTTTGCATTTCCTTATTACTAAGTTGCTTCATACCTTTAATTTGAATTTTC
ATAATAATCACCTCGCTTTTAGGATAATGTGTTTTTGAAGTACGGAGGAAT
TATTTCTATTAACGTACATTTTTTAAATTTAAACGTGAATCACCATGAAAT
TCCAACTATGCTATCAATCAATATTTGATGAATGTCAAAGTCACTATTTTG
AATAGATCTTGATTACTTTGAACATACTTAGAGGATAAAATAACATTAACT
TAAGTGTAGCGCTAAAGAATTCAAGATAAACTCCCATTAGTAACTGTACA
AAA

The similarity index of the five strains sequence to *plnA* sequence in NCBI was 98.8%.

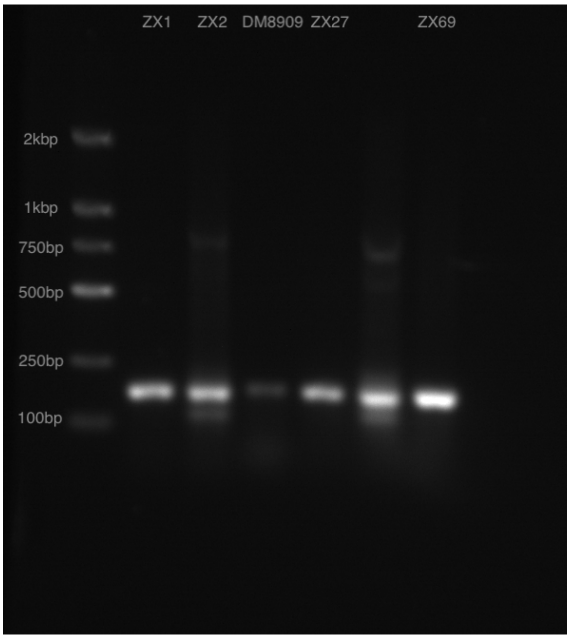

Supplementary Figure S2: The *plnB* gene detection results of 5 strains.

The sequencing results of the *plnB*(165bp) are the following:

nBsTnttdSsGATATCTGCCACTGTAACACCATGACTGAGAATAAAGTCAGTG
TTAGCTATTTCAATCGTCAACTTAACCCCGCACTGTTTCGCATAAAAAAAC
TTCTGTACAACTAACCCGCGCAATATCTCATTCTTCAGATGCtGAACACTT
GCAAAGCTACCGTCATTTAGGCTAGCTCTGAAAACAA

The similarity index of the five strains sequence to *plnB* sequence in NCBI was 91.0%.

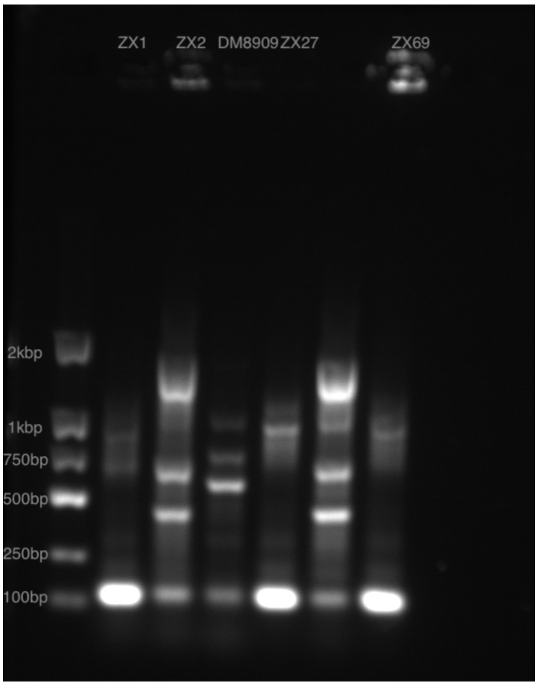

Supplementary Figure S3: The *plnC* gene detection results of 5 strains.
The sequencing results of the *plnC*(108bp) are the following:
TATCGATAATCCAACGGTGCAATCCGCCGCTCCAGTGTCAAAAAAGATAA
CTCTTCATGAGTTGTAATAAATACAATTTGTGCACAAGGTAACTGCTGCCG
AATTTCATCTGCTCGA
The similarity index of the five strains sequence to *plnC* sequence in NCBI was 96.5%.

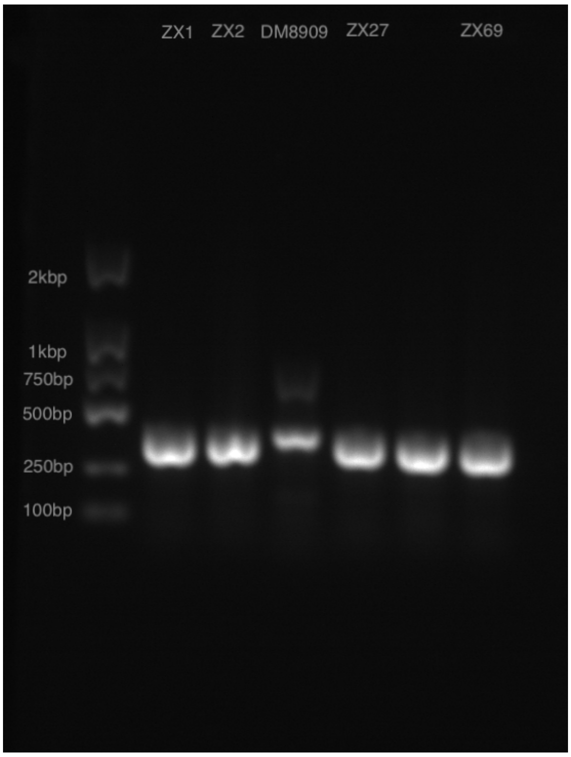

Supplementary Figure S4: The *plnD* gene detection results of 5 strains.
The sequencing results of the *plnD*(414bp) are the following:
TGCATCGGAAAAAATTGCGGATACTTTTCTTCGAGCGCATTTAAATTTCCT
GGGAACTCAGCAACCTTATTAATAGCATGGAGTTGTACGCTGCCCGGACG
CAGTTTAGATGTACTCAACAAAATCACATCATCTAATGCGAGTGAAAAGT
ATCGCGTTCCTAACTTATAGTTAAAAACATCTTTGCGCTGACTATTAGTCT
TTTTTAATTCGTTCTGTACTACATTGATGTCCTTAATAATCCTTTGCGTAAT
TAGGTCAGCAGACTGGTCTTTCAAAATATAATCCAACGGTGCAATCCGCC
GTTCCAGAGTTACAAACGATAGCTCATCGTGTGTTGTAATGAAAACTATTT
TAGCCAATGGTATCGTtGCCCGAaTtCgACTGGCTAATTCAAGTCCAGTCTG
TTTTGTCCTCAA
The similarity index of the five strains sequence to *plnD* sequence in NCBI was 98.1%.

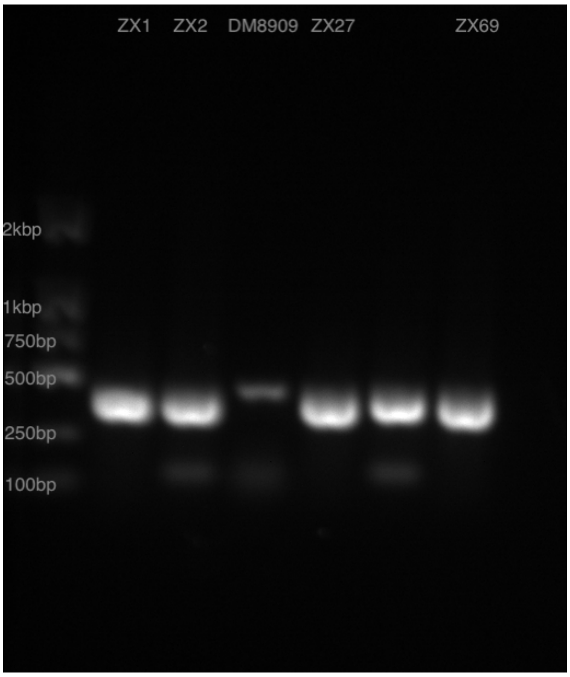

Supplementary Figure S5: The *plnEF* gene detection results of 5 strains.
The sequencing results of the *plnEF*(428bp) are the following:
TCAGGTTGCCCGCAAAAAAAGCTTGCCAAAATATCTGGTGGTTTTAATCG
GGGCGGTTATAACTTTGGTAAAAGTGTTCGACATGTTGTTGATGCAATTGG
TTCAGTTGCAGGCATTCGTGGTATTTTGAAAAGTATTCGTTAATTTTCTTG
GGGGAGATCAACAATTATGAAAAAATTTCTAGTTTTGCGTGACCGTGAAT
TAAATGCTATTTCAGGTGGCGTTTTCCATGCCTATAGCGCGCGTGGCGTTC
GGAATAATTATAAAAGTGCTGTTGGGCCTGCCGACTGGGTCATTAGCGCT
GTCCGAGGATTCATCCACGGATAGTTCAAGCCATCAAGTTTAAGCACTAT
AAGAAAGCACTCGATTTATGACTGGGCCTGCAGTGCTCAGCcTTTTTAGTT
TATATGGGGGGAATTTAAAACTATGCCC
The similarity index of the five strains sequence to *plnEF* sequence in NCBI was 99.4%.

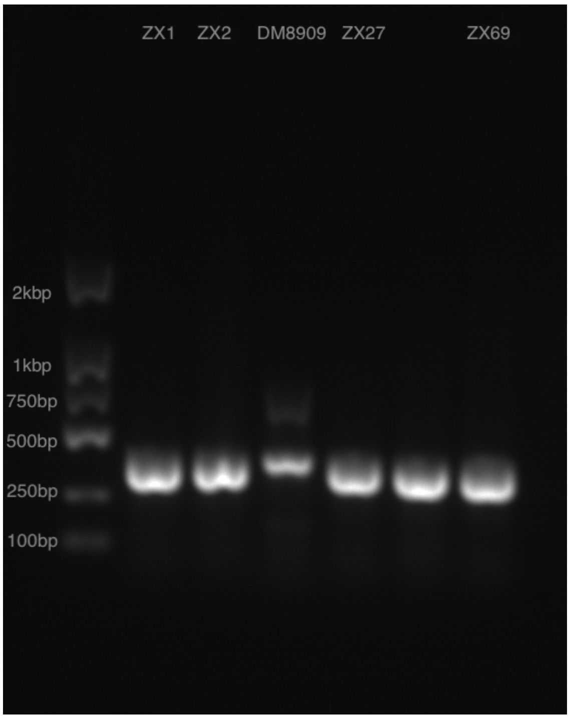

Supplementary FIGURE S6: The *plnI* gene detection results of 5 strains.
The sequencing results of the *plnI*(450bp) are the following:
GCTTCGTTTATCCTTCCTCTAAGCATTGGAAGTTaTTGAGTaTCcTAAaTATT
GaAGATAAAATAGACGAGTAATCATCCATTCTGAATTCCTATAAATTATTG
TTTGCAGCACTACAATTTCAATTATCTCTGAAGTTTTGGGTTACGCTAAGA
GCTAGTCATATCGCTAGCTAAATTTCACGTCTTAACGCTATGCTAATATAC
TTTACGATCGAGTACTGTGCTTACTTTTTAGTCATTTTAATACTCATAAGAT
CACAATTAGAGCCCTACTCCAACTATCTTCAGCGCCTATATTATATAATCA
GATTTCATATTCATAACAAAAGAGCCCTGTCAAATAACACTGACAGAAGC
TCTAAATAATATTAAAAAACTAGTTGTCTCTCAACAACTtATtCAATtCCCG
CGACTTTCTAAACGAAGCCTTACACCTAATTTACCCGTCGAGA
The similarity index of the five strains sequence to *plnI* sequence in NCBI was 96.1%.

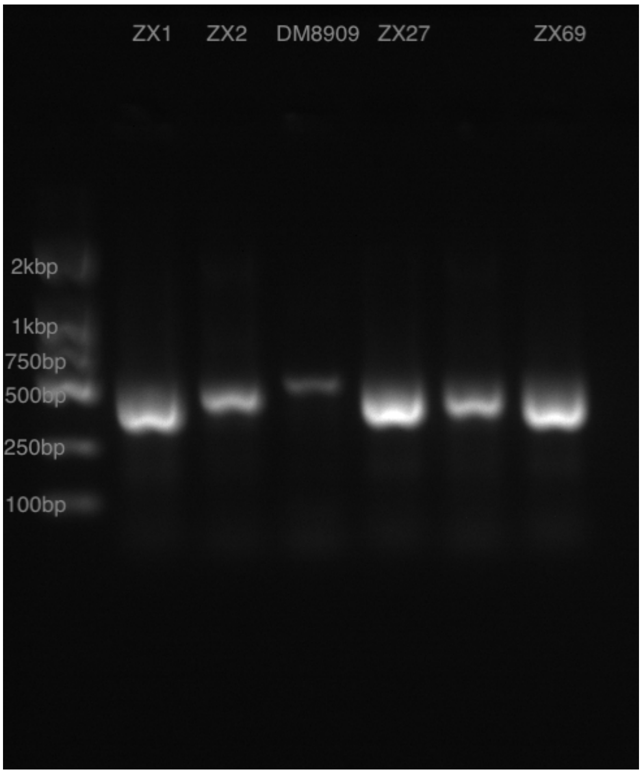

Supplementary Figure S7: The *plnJ* gene detection results of 5 strains.
The sequencing results of the *plnJ*(475bp) are the following:
TTAACGACGGGATGCTCTGCCAGCTTCGCCATCATAAAATCCTTTTCTTAA
ACTAGACCAGAAATTTTTCCAAGCGCCTCCCCCAACAACCCCGTTCAACTT
ATTATTAGAAATAGGTGCAAATGCATCTACTACATCCAAATCCTTAATCAT
TTTGTTCACAGTCATACCTCTCCCTTTACTGTAGCAGTTAAAAATGGTTCT
ATGCGCTACGTATTTTTAATGCACTTATTAATATACGCTACAATTTAAAAA
AAATTATTTTAAATTTGTTATCGTACTATTTAATCGATTTAACGTAACTTGA
AAGTTGAATGTCTGACGATCCCATAACATTAATGGGATTATTTTTGCTATG
ACGAATTGTTAAATTAAGTGCAATATTTTACCAAAGAAAACTTCACTTGGC
CTTTTTCATTtCAAGCTCTTACGTGGCGATTATTTAAATCTATGACTAATGT
GATATTCCCTTGATTAAA
The similarity index of the five strains sequence to *plnJ* sequence in NCBI was 98.8%.

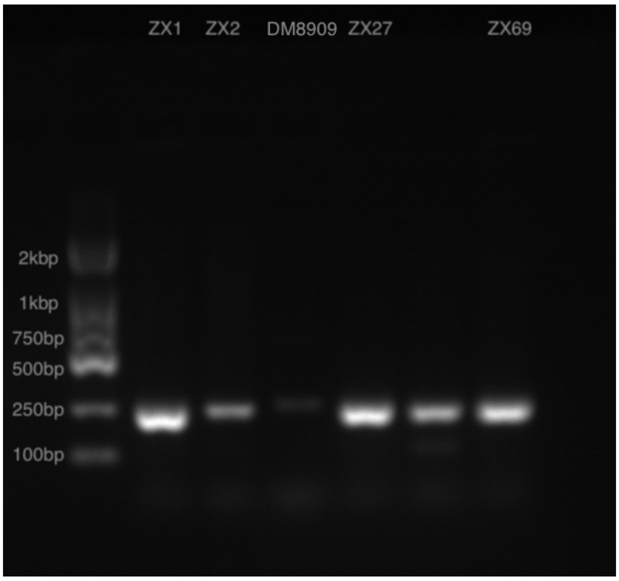

Supplementary **F**IGURE **S8**: The *plnK* gene detection results of 5 strains.
The sequencing results of the *plnK*(246bp) are the following:
TACTGCTGACGCTGAAAAGAATATTTCTGGTGGCCGTCGGAGTCGTAAAA
ATGGAATTGGATACGCTATTGGTTATGCGTTTGGCGCGGTTGAACGGGCC
GTGCTTGGTGGTTCAAGGGATTATAATAAGTGATAGAAATTGATTTCAAA
GAAAAATATTACCCGCTAGTGTTAGTGCTCTTTGTGGCTGGTTATATGGGT
GCGATCTTTCTTAATATTTATTTTGATTGGTTAGCAATGCTTACAGA
The similarity index of the five strains sequence to *plnK* sequence in NCBI was 97.7%.

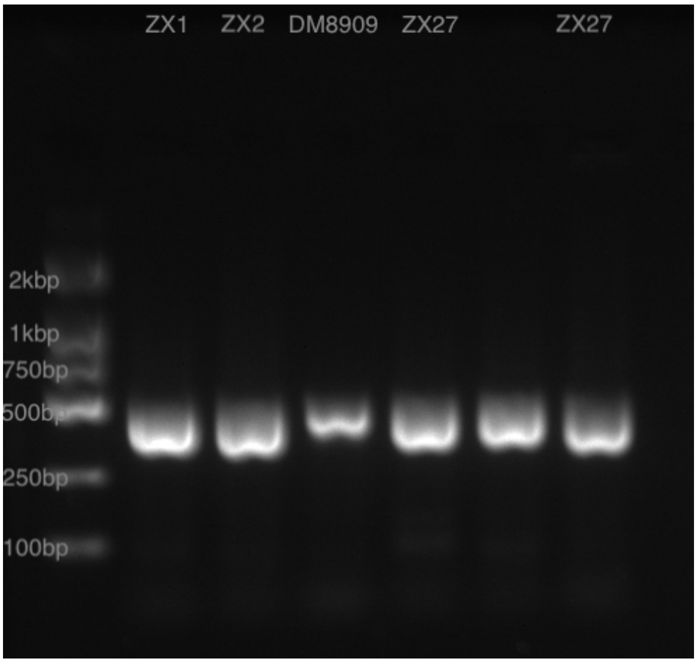

Supplementary **F**IGURE **S9**: The *plnG* gene detection results of 5 strains.
The sequencing results of the *plnG*(453bp) are the following:
CCTCGAAACCCATTCCCCCGTCCGCCGAGTGGCAAAAAAACTCATTGGCA
ATTCaAAGACGTGCCGAATATAGCCTAAAATAATTTCAATCGAAAGTCGTT

GACCTAAAACCGCCAATAAAAAATTCTGGGCATAAGTAAAGATAGCCTGA
AAGGTATAAAAAACGATTAGACCAATGGCCACCACTGCCAATGTACTGTG
CATATTATTGGGAATATAAGTATCAATCACCGCTTGTAAGAAATAAGACC
CACAGATACTAATAATCGTGATTAAAACTGCAGCAAGCACAATATTAATA
ACCAGTCGGCGCTGCTTTAATAAGCTTGGAATAAATCCCCATAGTGAGCC
CTTATCTTGCTTAACTGGCTTATATTCCGACTTCGGTGCAAAGAAAAGGGC
AACTCCGGACCATTCGCTTTCAAACCGTTCCTTTGACATACTGATTAACCG
CAA

The similarity index of the five strains sequence to *plnG* sequence in NCBI was 98.5%.

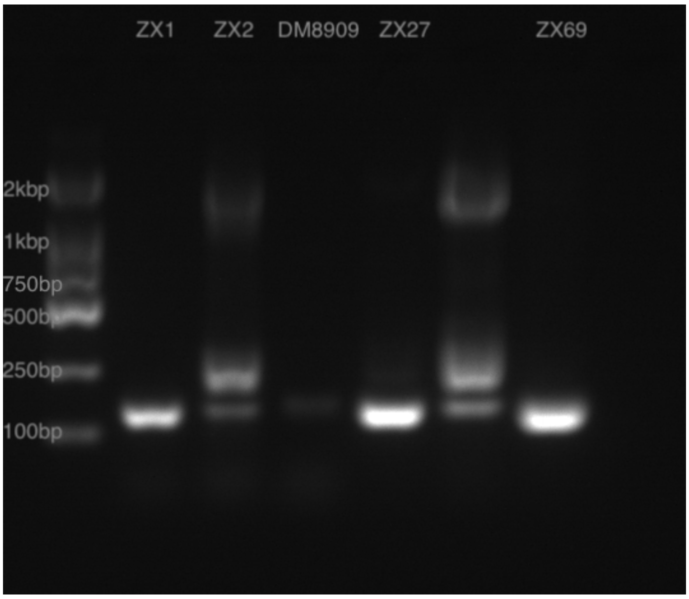

Supplementary Figure S10: The *plnN* gene detection results of 5 strains.

The sequencing results of the *plnN*(146bp) are the following:

TACCTAAACCATGCCATGCACTCGAAGTTCCCTCTGCTaCcTTTCccAGCaAT
GTAAGGCTCTTGTACCACCATGTCTTAGAATAGTTTTCCACCtTCAACGGT
AGTCAAATCTTTTTCTGCCATTTCGATACCTAACCCGGCAATAGA

The similarity index of the five strains sequence to *plnN* sequence in NCBI was 95.4%.

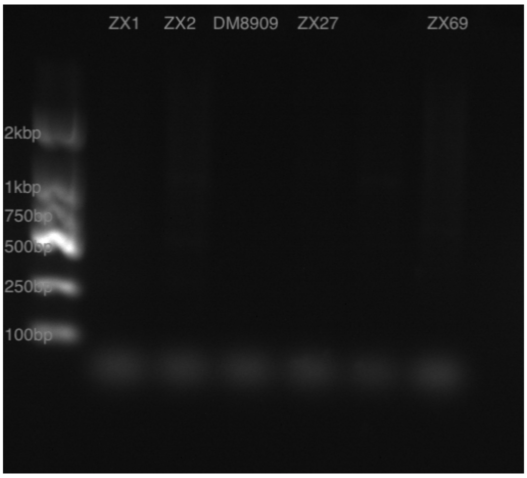

Supplementary FIGURE S11: The Plantaricin NC8 structural gene detection results of 5 strains.

The Plantaricin NC8 structural gene of 5 samples was negative.

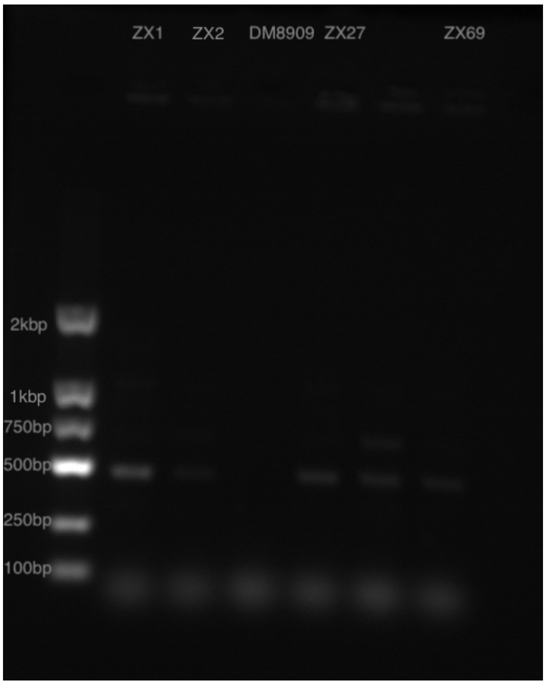

Supplementary FIGURE S12: Plantaricin S structural gene detection results of 5 strains.

The Plantaricin S structural gene of 5 samples was negative.

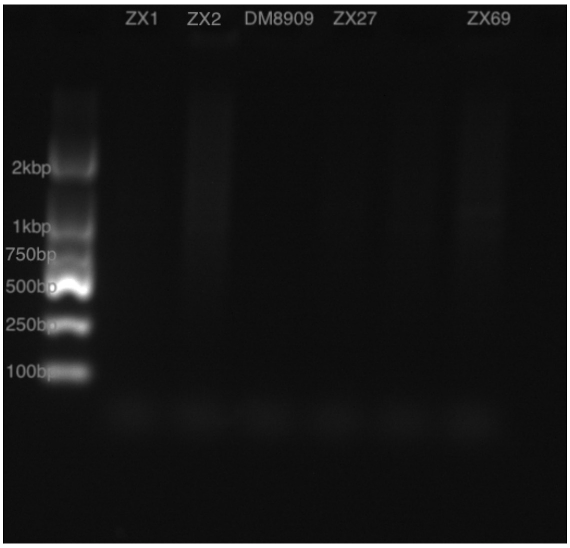

Supplementary FIGURE S13: The Plantaricin W structural gene detection results of 5 strains.

The Plantaricin W structural gene of 5 samples was negative.

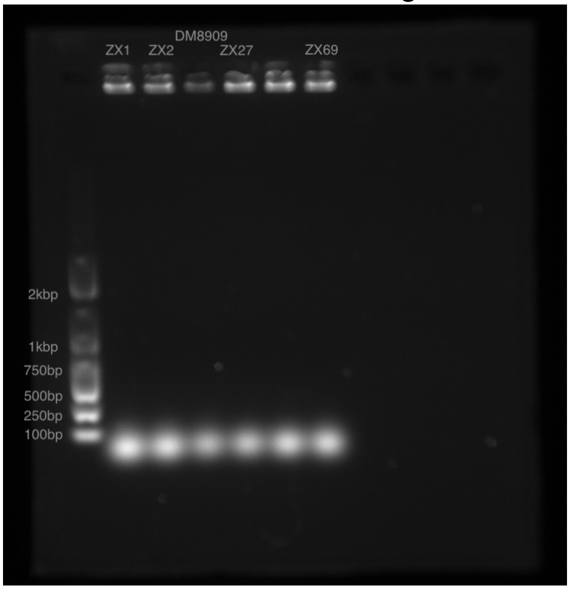

SupplementaryFIGURE S14: The *entA* gene detection results of 5 strains.

The *entA* gene of 5 samples was negative.

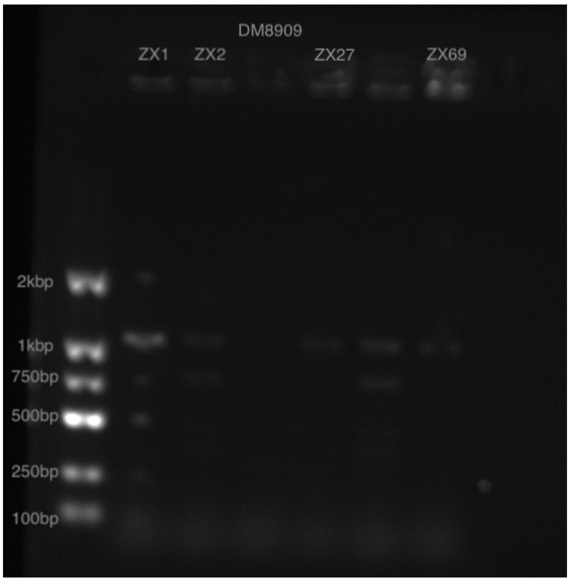

Supplementary **Figure S15**: The *gasA* gene detection results of 5 strains. The *gasA* gene of 5 samples was negative.

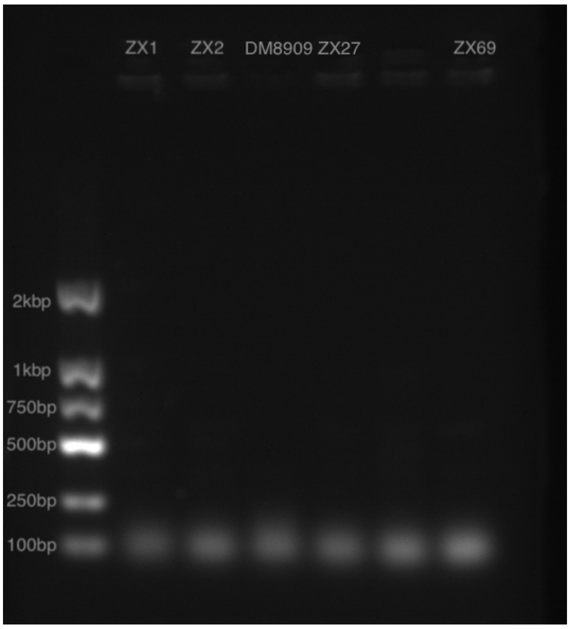

Supplementary **Figure S16**: The *laf* gene detection results of 5 strains. The *laf* gene of 5 samples was negative.
